# 

**Published:** 1887-09

**Authors:** 


					﻿VOL VII.
SEPTEMBER, 1887.
NO. 9.
THE
JiOMlEOPATHlC pH^lClAfl,
A MONTHLY JOURNAL OF MEDICAL SCIENCE.
" He is a freeman whom truth makes free, and all are slaves beside.'’
/"Seek the Truth: come whence it may, cost what it will."
EDITED BY
Edmund j. lee, m. d.,
AND
WALTER M. JAMES, M. D.
PHILADELPHIA :
No. 1183 SPRUCE 8TREET.
NEW YORK,
A. L. CHATTERTON & COMPANY, Publisher’s Agents.
londoni
ALFRED HEATH & CO., Sole Agents fob GREAT Britain,
114 Ebury Street, Eaton Square.
Yearly Subscription, $2.50, invariably in advance. Single numbers, 25 etc.
Entered at the Philadelphia Peet-OSce a* Second-Claw Kail Matter.
				

